# Ciprofloxacin resistance in community- and hospital-acquired *Escherichia coli* urinary tract infections: a systematic review and meta-analysis of observational studies

**DOI:** 10.1186/s12879-015-1282-4

**Published:** 2015-11-25

**Authors:** Oyebola Fasugba, Anne Gardner, Brett G. Mitchell, George Mnatzaganian

**Affiliations:** Faculty of Health Sciences, Australian Catholic University, 223 Antill Street, Watson, Australian Capital Territory 2602 Australia; Faculty of Arts, Nursing and Theology, Avondale College of Higher Education, 185 Fox Valley Road, Wahroonga, New South Wales 2076 Australia; School of Allied Health, Faculty of Health Sciences, Australian Catholic University, 17 Young Street, Fitzroy, Victoria 3065 Australia

**Keywords:** Antimicrobial resistance, Escherichia coli, Urinary tract infection, Systematic review, Meta-analysis

## Abstract

**Background:**

During the last decade the resistance rate of urinary *Escherichia coli* (*E. coli*) to fluoroquinolones such as ciprofloxacin has increased. Systematic reviews of studies investigating ciprofloxacin resistance in community- and hospital-acquired *E. coli* urinary tract infections (UTI) are absent. This study systematically reviewed the literature and where appropriate, meta-analysed studies investigating ciprofloxacin resistance in community- and hospital-acquired *E. coli* UTIs.

**Methods:**

Observational studies published between 2004 and 2014 were identified through Medline, PubMed, Embase, Cochrane, Scopus and Cinahl searches. Overall and sub-group pooled estimates of ciprofloxacin resistance were evaluated using DerSimonian-Laird random-effects models. The I^2^ statistic was calculated to demonstrate the degree of heterogeneity. Risk of bias among included studies was also investigated.

**Results:**

Of the identified 1134 papers, 53 were eligible for inclusion, providing 54 studies for analysis with one paper presenting both community and hospital studies. Compared to the community setting, resistance to ciprofloxacin was significantly higher in the hospital setting (pooled resistance 0.38, 95 % CI 0.36-0.41 versus 0.27, 95 % CI 0.24-0.31 in community-acquired UTIs, *P* < 0.001). Resistance significantly varied by region and country with the highest resistance observed in developing countries. Similarly, a significant rise in resistance over time was seen in studies reporting on community-acquired *E. coli* UTI.

**Conclusions:**

Ciprofloxacin resistance in *E. coli* UTI is increasing and the use of this antimicrobial agent as empirical therapy for UTI should be reconsidered. Policy restrictions on ciprofloxacin use should be enhanced especially in developing countries without current regulations.

**Electronic supplementary material:**

The online version of this article (doi:10.1186/s12879-015-1282-4) contains supplementary material, which is available to authorized users.

## Background

Urinary tract infections (UTI) are one of the most frequent bacterial infections affecting people both in the community and in hospitals [1]. It is estimated that about 150 million people per annum are diagnosed with UTI worldwide [2]. A recent World Health Organisation (WHO) report on antimicrobial resistance (AMR) surveillance specified nine bacteria of international concern which are responsible for some of the most common infections in community and hospital settings [3]. *Escherichia coli* (*E. coli)*, the pathogen most often implicated in UTIs, is listed as one of the nine. In all six WHO regions (Africa, Americas, Eastern Mediterranean, European, South-East Asia and Western Pacific) high rates of antimicrobial resistance have been observed in this pathogen [3].

Ciprofloxacin is the most commonly prescribed fluoroquinolone for UTIs because it is available in oral and intravenous preparations [4]. It is well absorbed from the gastrointestinal tract after oral administration. It also has a documented safety profile, broad Gram negative organism coverage and high urinary excretion rate [4]. During the last decade the resistance rate of *E. coli* to fluoroquinolones such as ciprofloxacin has increased [5]. A 10 year analysis of urinary *E. coli* specimens in Switzerland, found an increasing trend in resistance to ciprofloxacin, from 1.8 to 15.9 % [6]. Fluoroquinolones are ranked as one of four of the highest priority critically important antimicrobials [7] as they have an important role in the treatment of more severe infections, such as septicaemia. Therefore resistance to fluoroquinolones can have serious clinical consequences. They are one of few available therapies for serious *Salmonella* spp. and *E.coli* infections [5]. Resistance to fluoroquinolones emerges quickly, and this is likely to be related to the biology of resistance as well as a direct response to drug pressure [8]. They should therefore be used with caution and reserved for severe infections, and preceded by antimicrobial susceptibility testing of the bacteria involved [5]. The most recent Infectious Diseases Society of America (IDSA) guidelines recommend that fluoroquinolones should be reserved for important uses due to their propensity for ecological unfavorable effects of antimicrobial therapy such as the selection of drug-resistant pathogens and colonisation or infection with multidrug-resistant organisms [9].

Recent prescribing guidelines recommend reserving ciprofloxacin use for more severe infections and resistance to this agent is increasing prompting further research in this area [6, 10, 11]. Published quantitative syntheses of overall ciprofloxacin-resistant *E. coli* UTI prevalence and incidence in hospital and community settings are absent. This systematic review of observational studies therefore aims to compare ciprofloxacin resistance in both settings. Knowledge about ciprofloxacin resistance in community- and hospital-acquired *E. coli* UTIs will provide information for control of resistant pathogens. This review also has the potential to provide a basis for which future interventions can be evaluated. The findings will, in addition, make available information on ciprofloxacin resistance in various regions of the world providing some evidence for further regulatory control of ciprofloxacin use globally.

## Methods

### Protocol and registration

The protocol for conducting this review has been registered and can be accessed on the International prospective register of systematic reviews (PROSPERO) (available at http://www.crd.york.ac.uk/prospero/ with registration number: CRD42014014473). Prior to registration, the protocol was reviewed by a reviewer external to the study team. Ethics approval was not sought as this review synthesized data from published studies for which approval had already been obtained.

### Search strategy

We conducted a systematic review of observational (cross sectional, cohort and case control) studies published in the last 11 years (2004–2014) reporting on ciprofloxacin resistance in community- and hospital-acquired *E. coli* UTIs. This time limit is based on changes in the microbiology and epidemiology of antimicrobial resistant pathogens which occurred in the past decade with subsequent changes in treatment regimens and patient outcomes [12]. Reporting of this review complied with the preferred reporting items for systematic reviews and meta-analyses (PRISMA) [13].

The electronic bibliographic databases MEDLINE/PubMed, EMBASE, Cochrane, CINAHL and Scopus were searched. Searches were conducted for words in the title or abstract or within the full text of the papers. These included both keywords only and keywords with medical subject headings (MeSH) using the search terms ‘resistance’, ‘urinary tract infection’ and ‘Escherichia coli’ from 1st January 2004 to 31st December 2014 (see Additional file 1). The reference lists of papers identified from the electronic databases were hand-searched for additional papers.

### Inclusion and exclusion criteria

Papers were included if they reported prevalence or incidence rates of ciprofloxacin resistance in community- or hospital-acquired *E. coli* UTIs. Papers reporting on urinary *E. coli* ciprofloxacin susceptibility in which resistance rate could be calculated were also included. We included papers involving adults and/or children. Only peer reviewed manuscripts were considered. Grey material which includes unpublished literature, conference abstracts, letters to editors, newsletters and reports were excluded. Non-peer reviewed literature were also excluded. Papers written in languages other than English were also excluded. In addition, papers not clearly specifying the setting (hospital-acquired or community-acquired); drug (ciprofloxacin) or sample (urine) were excluded. Papers that focused on specific sub-populations (e.g. diabetics and patients with recurrent UTI) were also excluded as these did not represent the general population. This review included only papers that used the Centers for Disease Control and Prevention (CDC) definition of microbiologically confirmed UTI (≥10^5^ colony forming unit/ml) [14].

#### Definitions

For the purpose of this review, a study was defined as all data from a published paper with the only distinction being ‘hospital’ or ‘community’ setting. Therefore, if a single paper meeting the eligibility criteria reported data on both settings, they were included as two separate studies.

Community-acquired UTI was defined as positive samples obtained from (i) outpatient clinics; (ii) general practice (GP) clinics; (iii) emergency departments; (iv) within 48 h of hospital admission or (v) from nursing homes or residential aged care facilities [15–17].

Hospital-acquired UTI was defined as positive samples obtained (i) after 48 h of hospital admission or (ii) within 48 h of hospital discharge [15].

Important changes in healthcare delivery over the last few years have seen some usually inpatient procedures now more often than not performed on an outpatient basis [18]. Patients transition freely within sometimes loosely defined levels of the health care system, for example between long-term care or rehabilitation services, to acute-care centres [19, 20]. This study only considered hospital-acquired UTIs as opposed to a wider definition of healthcare associated UTIs, to avoid this confusion.

### Study selection

The titles and abstracts of all papers identified in the electronic databases were examined and assessed for relevance and appropriateness to the principal objective of the systematic review. Irrelevant studies were excluded. Full texts of the potentially relevant papers were printed and carefully assessed against the systematic review inclusion and exclusion criteria. Those not meeting the criteria were excluded. The remaining papers deemed to have data relevant to the systematic review and meta-analysis were assessed for quality and risk of bias.

The study selection process and other stages of the review were performed by the lead author (OF). At each stage, 10 % of papers identified were also screened against the study criteria independently by other authors (AG, GM and BM). Discrepancies in either the application of inclusion or exclusion of papers, quality assessment or on data extraction were discussed among all authors to make the final decision.

### Data extraction process

Data were extracted by one author (OF) and 10 % of papers eligible for data extraction were independently extracted by another author (AG). Data extraction was compared between AG and OF demonstrating 100 % agreement for all items except the study design. This variable was therefore assessed by all authors. Where there was missing information on the study design of papers to be included in the meta-analysis, attempts were made to contact the authors. When there was no response, consensus on the study design was reached by all authors. Agreement between authors was assessed using Kappa coefficient. The agreement between all authors in deciding on the study design was 71 % (Kappa (95 % CI) = 0.429 (0.154–0.703), *P* Value = 0.003). Papers for which no agreement could be reached on the design, based on insufficient information, were assigned as non-classifiable. Any other missing information in the included papers was recorded as ‘not stated’.

The first author, year of study, country of study, study setting, age and sex distribution, co-morbidities, sample size, study design, study aim, antimicrobial susceptibility testing method, ciprofloxacin resistance rate, risk factors for ciprofloxacin resistance (i.e. previous antibiotic use) and mortality data (if reported) were extracted. Where the ciprofloxacin resistance rate was not available, the susceptibility rate was used to determine resistance.

### Risk of bias in individual studies

Quality and risk of bias of the final papers included in the review was conducted using a modified version of the Newcastle-Ottawa Scale (NOS) which is a risk of bias assessment tool for observational studies recommended by the Cochrane Collaboration [21, 22]. Content validity and inter-rater reliability of this tool have been established [22]. Studies were rated by assigning a judgment of ‘Low risk’ of bias, ‘High risk’ of bias, or ‘Unclear risk’ of bias according to published criteria [21].

### Statistical analysis

Pooled ciprofloxacin resistance proportions (with 95 % confidence intervals) in patients with *E. coli* UTI were separately calculated and compared between hospital and community settings using a random-effects meta-analysis model based on DerSimonian and Laird method [23, 24]. This method incorporates an estimate of the between-study variation into both the study weights and the standard error of the estimate of the common effect. The precision of an estimate from each included study was represented by the inverse of the variance of the outcome pooled across all studies. If the value of the pooled prevalence was within the 95 % CI, then the effect size was statistically significant at the 5 % level (*P* < 0.05). The heterogeneity among studies was assessed by using the I^2^ statistic with a *P* value of <0.05 considered statistically significant, and I^2^ values below 25 % indicating low heterogeneity, 25–75 % moderate heterogeneity and over 75 % high heterogeneity [25]. Subgroup analyses were done by risk of bias, study duration, age group, UTI symptoms, world region and economy of country (categorised as developed and developing using the World Bank classification [26]). A meta-regression analysis was used to determine the effect of measured covariates on the observed heterogeneity in resistance estimates across studies [23]. Assessment of publication bias was estimated using funnel plots. Further analysis was undertaken to examine pooled ciprofloxacin resistance over time using the median study year. For studies occurring over 2 years, the first year was used; for studies occurring over 4 years, the 2nd year was used; for those over 6 years, the 3rd year was used. The non-parametric Spearman’s rho correlation coefficient was calculated to determine significance in resistance trend over time. Statistical analyses were undertaken using Stata statistical softwareversion 13 [27].

## Results

### Study selection

**Fig. 1:**
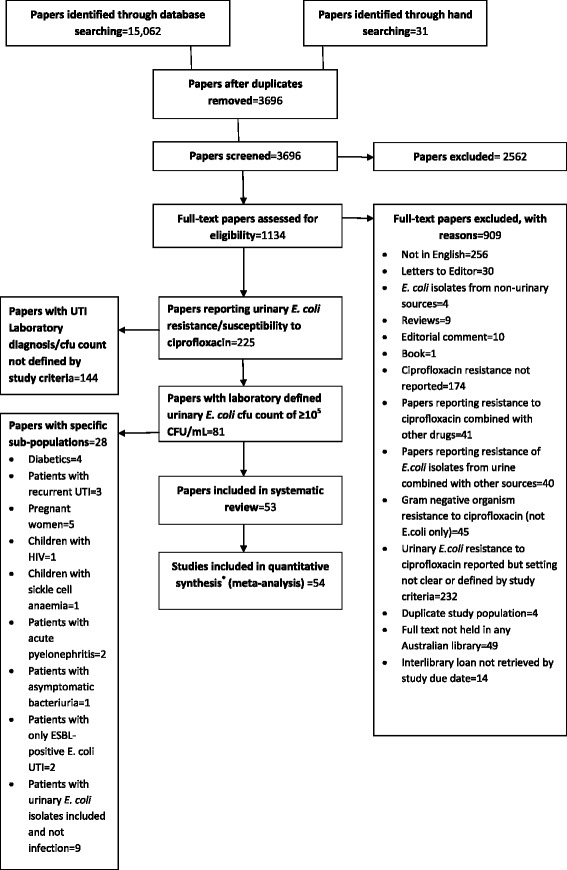
PRISMA flow diagram of study selection. (*54 studies from 53 papers)

Electronic database searches identified 15,062 potential studies and 31 additional studies were identified through hand searching. After 11,397 duplicates were removed, 3696 articles remained for title and abstract screening. We assessed 1134 as potentially eligible and retrieved the full text of these articles. After applying inclusion and exclusion criteria, 53 papers (5 %) were deemed to have data relevant to the systematic review and meta-analysis. These 53 papers consisted of 54 studies comprising three hospital-acquired *E. coli* UTI studies and 51 community-acquired *E. coli* UTI studies. There was one paper that compared resistance in both hospital and community settings hence reported as two studies [15]. The PRISMA flow chart describing the papers identified from the search strategy and reasons for exclusion is shown in Fig. 1.

### Study characteristics

Geographically, 53 of the 54 studies were carried out in Asia (28 %; *n* = 15), Europe (24 %; *n* = 13), Middle East (15 %; *n* = 8), Africa (13 %; *n* = 7), North America (11 %; *n* = 6) and South America (7 %; *n* = 4). The remaining study was conducted in multiple countries [28]. There were 17 (31 %) studies conducted in developed countries and 36 (67 %) in developing countries. The majority of the studies (80 %) followed a cross sectional design. The duration of studies ranged from 2 months to 84 months (median  = 15.5; IQR = 12.0-30.0). The mean age and sex proportion of patients with an *E. coli* UTI were stated in 13 % (*n* = 7) and 44 % (*n* = 24) of studies respectively. Most study populations included patients of both sexes although 19 % (*n* = 10) included only women. Antimicrobial susceptibility testing and interpretation was performed using the disk diffusion method (74 %) and Clinical and Laboratory Standards Institute (CLSI) criteria (83 %) respectively in most studies. Table 1 provides further details on the description of the included studies.

**Table 1:** Description of studies included in meta-analysis

Study author	Country	Design^a^	Setting	Risk of bias	Study duration^b^ (months)	Number of positive *E. coli* UTI samples^c^	Number of ciprofloxacin resistant *E. coli*	Proportion resistant (95 % CI)	Standard error	Weight^d^ (%)
Ahmad, 2012	India	Cross sectional	Community	Unclear	24	318	48	0.15 (0.11, 0.19)	0.02	2.09
Akoachere et al., 2012	Cameroon	Cross sectional	Community	Low	12	43	11	0.26 (0.13, 0.39)	0.07	1.61
Akram et al., 2007	India	Cross sectional	Community	High	12	61	42	0.69 (0.57, 0.80)	0.06	1.70
AlSweih et al., 2005	Kuwait	Cross sectional	Community	High	12	1535	81	0.05 (0.04, 0.06)	0.01	2.15
Al-Tawfiq et al., 2009	Saudi Arabia	Cohort	Community	High	12	2281	592	0.26 (0.24, 0.28)	0.01	2.14
Ansbach et al., 2013	USA	Cross sectional	Community	High	7	98	2	0.02 (−0.01, 0.05)	0.01	2.12
Arabi et al., 2013	Iran	Cross sectional	Community	Low	33	103	23	0.22 (0.14, 0.30)	0.04	1.91
Araujo et al., 2011	Brazil	Cross sectional	Community	Unclear	24	391	36	0.09 (0.06, 0.12)	0.01	2.12
Arslan et al., 2005	Turkey	Cross sectional	Community	Low	5	514	135	0.26 (0.22, 0.30)	0.02	2.09
Astal, 2005	Palestine	Cross sectional	Community	High	6	252	30	0.12 (0.08, 0.16)	0.02	2.09
Azap et al., 2010	Turkey	Cohort	Community	Unclear	12	464	139	0.30 (0.26, 0.34)	0.02	2.08
Bahadin et al., 2011	Singapore	Cross sectional	Community	Unclear	12	90	22	0.24 (0.16, 0.33)	0.05	1.86
Biswas et al., 2006	India	Cross sectional	Community	High	36	354	124	0.35 (0.30, 0.40)	0.03	2.05
Bouchillon et al., 2013	USA	Cross sectional	Community	High	24	723	234	0.32 (0.29, 0.36)	0.02	2.10
Bouchillon et al., 2013	USA	Cross sectional	Hospital	High	24	253	103	0.41 (0.35, 0.47)	0.03	11.83
Dash et al., 2013	India	Cross sectional	Community	Low	30	397	212	0.53 (0.48, 0.58)	0.03	2.05
Dimitrov et al., 2004	Kuwait	Cross sectional	Community	High	84	780	92	0.12 (0.10, 0.14)	0.01	2.13
Farshad et al., 2011	Iran	Cross sectional	Community	Low	12	90	8	0.09 (0.03, 0.15)	0.03	2.01
Ghadiri et al., 2012	Iran	Cross sectional	Hospital	High	24	200	80	0.40 (0.33, 0.47)	0.03	9.41
Gobernado et al., 2007	Spain	Cross sectional	Community	Low	12	2292	418	0.18 (0.17, 0.20)	0.01	2.14
Ho et al., 2010	Hong Kong	Cross sectional	Community	Low	24	271	35	0.13 (0.09, 0.17)	0.02	2.09
Hoban et al., 2011	Multiple countries	Cross sectional	Hospital	High	24	1643	624	0.38 (0.36, 0.40)	0.01	78.76
Ismaili et al., 2011	Belgium	Cohort	Community	High	24	189	5	0.03 (0.00, 0.05)	0.01	2.13
Kashef et al., 2010	Iran	Cross sectional	Community	High	30	578	180	0.31 (0.27, 0.35)	0.02	2.09
Kiffer et al., 2007	Brazil	Cross sectional	Community	Unclear	48	22679	2699	0.12 (0.11, 0.12)	0.002	2.15
Killgore et al., 2004	USA	Case–control	Community	Low	12	120	40	0.33 (0.25, 0.42)	0.04	1.89
Kimando et al., 2010	Kenya	Cross sectional	Community	Unclear	6	92	6	0.07 (0.01, 0.12)	0.03	2.05
Kothari et al., 2008	India	Cross sectional	Community	High	6	361	260	0.72 (0.67, 0.77)	0.02	2.06
Kurutepe et al., 2005	Turkey	NC	Community	High	72	880	174	0.20 (0.17, 0.22)	0.01	2.12
Lau et al., 2004	Taiwan	Cross sectional	Community	Unclear	13	80	14	0.17 (0.09, 0.26)	0.04	1.89
Ljuca et al., 2010	Bosnia & Herzegovina	Cross sectional	Community	High	36	43	4	0.09 (0.01, 0.18)	0.04	1.87
Longhi et al., 2012	Italy	NC	Community	Low	6	154	36	0.23 (0.17, 0.30)	0.03	1.98
Martinez et al., 2012	Colombia	Cross sectional	Community	High	2	102	39	0.38 (0.29, 0.48)	0.05	1.83
Miragliotta et al., 2008	Italy	Cohort	Community	Low	60	2589	422	0.16 (0.15, 0.18)	0.01	2.14
Molina-Lopez et al., 2011	México	Cross sectional	Community	High	48	119	65	0.55 (0.46, 0.64)	0.05	1.86
Moreira et al., 2006	Brazil	Cross sectional	Community	Unclear	15	544	65	0.12 (0.09, 0.15)	0.01	2.12
Murugan et al., 2012	India	Cohort	Community	High	12	204	144	0.71 (0.64, 0.77)	0.03	2.00
Muvunyi et al., 2011	Rwanda	Cross sectional	Community	Low	6	72	23	0.32 (0.21, 0.43)	0.05	1.75
Mwaka et al., 2011	Uganda	Cross sectional	Community	High	NS	27	9	0.33 (0.16, 0.51)	0.09	1.32
Ni Chulain et al., 2005	Ireland	Cross sectional	Community	High	5	723	18	0.02 (0.01, 0.04)	0.01	2.15
Olson et al., 2012	USA	Cross sectional	Community	Unclear	16	95	4	0.04 (0.00, 0.08)	0.02	2.08
Otajevwo, 2013	Nigeria	Cross sectional	Community	High	6	5	4	0.80 (0.45, 1.15)	0.18	0.63
Prakash et al., 2013	India	Cross sectional	Community	Low	NS	23	16	0.70 (0.51, 0.88)	0.10	1.26
Randrianirina et al., 2007	Madagascar	Cross sectional	Community	Low	28	607	100	0.16 (0.14, 0.19)	0.02	2.12
Rani et al., 2011	India	Cross sectional	Community	Unclear	6	208	151	0.73 (0.67, 0.79)	0.03	2.01
Shaifali et al., 2012	India	Cross sectional	Community	Unclear	12	46	28	0.61 (0.47, 0.75)	0.07	1.54
Shariff et al., 2013	India	Cross sectional	Community	High	18	491	160	0.33 (0.28, 0.37)	0.02	2.08
Sire et al., 2007	Senegal	Cross sectional	Community	Low	33	1010	157	0.16 (0.13, 0.18)	0.01	2.13
Sood et al., 2012	India	NC	Community	High	30	214	160	0.75 (0.69, 0.81)	0.03	2.02
Stratchounski et al., 2006	Russia	NC	Community	Low	48	423	18	0.04 (0.02, 0.06)	0.01	2.14
Vellinga et al., 2012	Ireland	Case–control	Community	Low	9	633	78	0.12 (0.10, 0.15)	0.01	2.12
Wang et al., 2014	China	Cross sectional	Community	High	8	129	91	0.71 (0.63, 0.78)	0.04	1.92
Yildirim et al., 2010	Turkey	Cross sectional	Community	Unclear	24	450	85	0.19 (0.15, 0.23)	0.02	2.10
Yolbas et al., 2013	Turkey	Cross sectional	Community	High	12	113	24	0.21 (0.14, 0.29)	0.04	1.93

^a^Non-classifiable design

^b^Not stated

^c^Study denominator

^d^Weights are from random effects analysis using DerSimonian-Laird model

### Pooled ciprofloxacin resistance

**Fig. 2:**
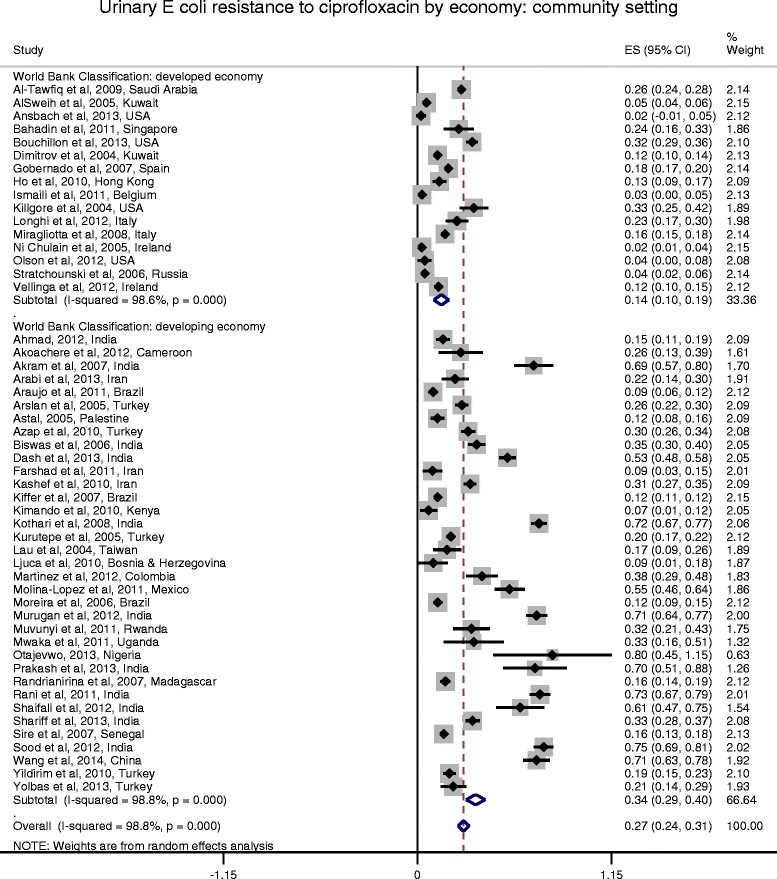
Forest plot of ciprofloxacin resistance in community-acquired *E. coli* UTI by economy

**Fig. 3:**
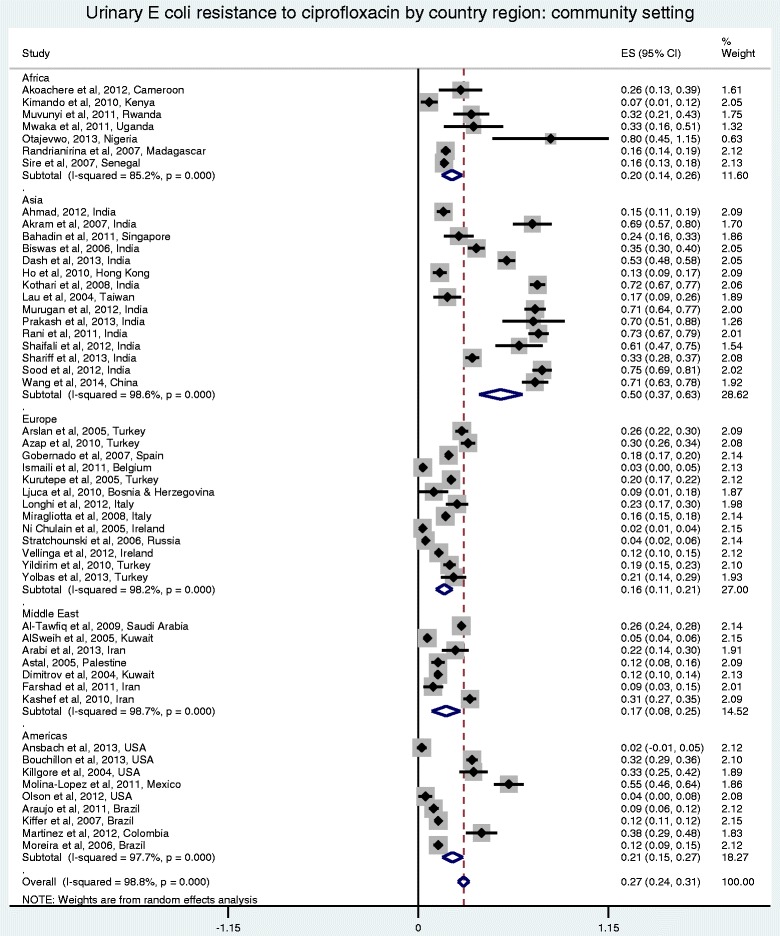
Forest plot of ciprofloxacin resistance in community-acquired *E. coli* UTI by region

**Fig. 4:**
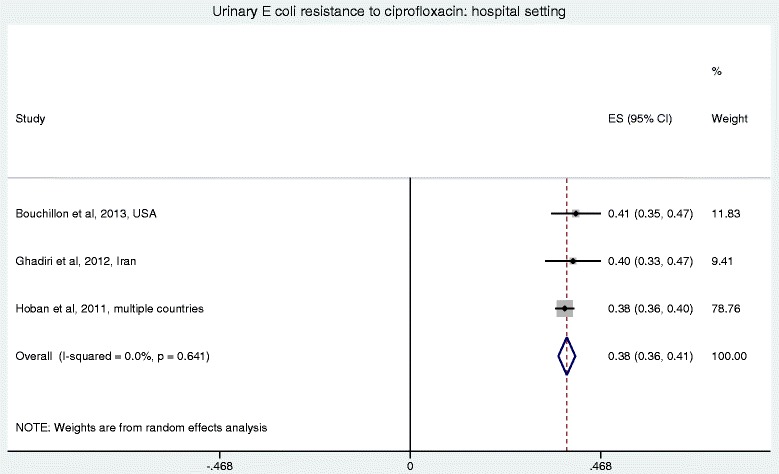
Forest plot of ciprofloxacin resistance in hospital-acquired *E. coli* UTI

Figures 2 and 3 show the forest plots of studies reporting on ciprofloxacin resistance in community acquired *E. coli* UTI by region and economy, respectively. Figure 4 shows the forest plot of studies reporting on ciprofloxacin resistance in hospital acquired *E. coli* UTI. Compared with the community-setting, resistance to ciprofloxacin in *E coli* UTIs was significantly higher in the hospital-setting (*P* < 0.001). Overall, the pooled rate for ciprofloxacin resistance in patients with community-acquired *E. coli* UTIs was 0.27 (95 % CI: 0.240-0.310), compared with 0.38 (95 % CI: 0.360-0.410) in the hospital setting. There was substantial heterogeneity among the community-setting studies (*I*^2^ = 98.8 %, *P* < 0.0001), but very little in the hospital ones (*I*^2^ = <0.010 %, *P* = 0.641). Further analysis of studies reporting on community-acquired *E. coli* UTI by region (Fig. 3) showed that Asia had the highest pooled resistance. Analysis by economy based on the World Bank classification (Fig. 4) showed a higher pooled resistance in developing countries.

### Resistance over time in community-acquired UTI studies

**Fig. 5:**
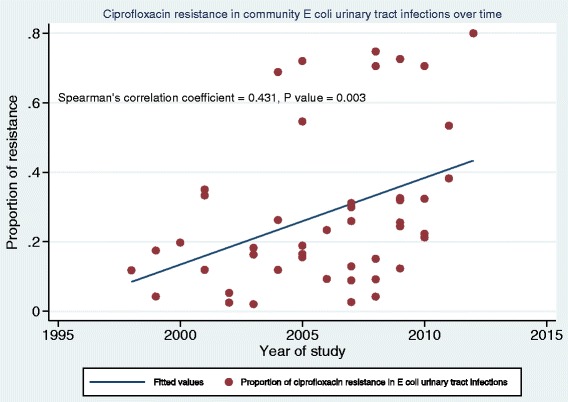
Scatter plot of ciprofloxacin resistance in community-acquired UTI by year of study (1998–2012). *N* = 47 (4 studies excluded due to missing information on year study was conducted)

Figure 5 shows the scatter plot of ciprofloxacin resistance in 47 studies reporting on community-acquired UTI using the median study year for each study. Four studies did not provide data on the year(s) the study was conducted and were excluded from this analysis [29–32]. The results of the Spearman’s rho correlation test showed a statistically significant rise in resistance over time (*n* = 47, *r*_*s*_ = 0.431, *P* = 0.003). Similar findings were observed for developing countries. There was no significant rise in resistance over time in developed countries.

### Subgroup analyses

Sub-group analysis was conducted within each major setting. For community-acquired UTI studies (Table 2), there was a significant difference in the pooled resistance within each subgroup examined (risk of bias, study duration, economy, region, age group and UTI symptoms). The subgroup analyses results for studies reporting on hospital-acquired *E. coli* UTI (see Additional file 2) showed no difference in the pooled resistance within the subgroups examined (region, economy and UTI symptoms). When both settings were compared (see Additional file 3), there were significant differences noted for risk of bias (high), study duration (>12 months), economy (developed), region (Americas), age group (adults and children) and UTI symptoms (*P* < 0.001). There were no data available on mortality for comparison between settings.

**Table 2:** Subgroup analyses of pooled ciprofloxacin resistance in community setting

Subgroup		Community Setting *N* = 51	*P* value*
		Pooled resistance	
Risk of bias	Low and unclear *n* = 28 studies	0.221	<0.0001
	High *n* = 23 studies	0.337	
Study duration^a^	≤12 months *n* = 25 studies	0.323	<0.0001
	>12 months *n* = 24 studies	0.219	
Economy	Developed *n* = 16 studies	0.141	<0.0001
	Developing *n* = 35 studies	0.345	
Region	Africa, Asia and Middle East *n* = 29 studies	0.361	<0.0001
	Europe, North and South America *n* = 22 studies	0.174	
Age group^a^	Adults and children ^b^ *n* = 24 studies	0.265	<0.0001
	Adults only *n* = 19 studies	0.302	
UTI symptoms	Symptomatic and asymptomatic patients *n* = 11 studies	0.185	<0.0001
	Symptomatic patients only *n* = 40 studies	0.295	

*n* = number of studies reporting on community acquired UTI

*Comparing pooled resistance for difference in subgroup in community setting

^a^Studies with missing information on this sub-analysis were not included

^b^Studies reporting resistance in adults and children or children only

### Meta-regression analyses

Random effects meta-regression analyses of studies reporting on community-acquired *E. coli* UTI showed that country’s economy (*P* = 0.008), Asia as a region (*P* = 0.002), high risk of bias (*P* = 0.003), year of study (*P* = 0.020) and studies using only children as the study population (*P* = 0.030) were the study factors significantly accounting for the observed heterogeneity, responsible for 61 % of the between study variance (Adjusted R^2^) in ciprofloxacin resistance.

### Risk of bias

When studies were assessed for risk of bias using the Newcastle-Ottawa scale, 30 % (*n* = 16) were assessed as having a low risk of bias; 22 % (*n* = 12) unclear risk of bias and 48 % (*n* = 26) were deemed to have a high risk of bias. Further analysis of the 16 low risk studies only was consistent with findings reported from the analysis of all studies. An increasing resistance trend over time was also observed, however this increase did not reach statistical significance because of reduced statistical power.

## Discussion

The findings of this systematic review and meta-analysis highlight the higher ciprofloxacin resistance in hospital-acquired *E.coli* UTI when compared to community-acquired UTI. There is also substantial evidence that ciprofloxacin resistance in community-acquired *E. coli* UTI has been increasing in recent years. Resistance was also found to be significantly higher in developing countries reporting on *E. coli* UTI in community settings.

Antimicrobial resistance has been described as an international hazard to public health threatening the successful prevention and treatment of bacterial, viral, parasitic and fungal infections [3, 33]. As such, research into its prevention and reduction is very important. Our estimated pooled ciprofloxacin resistance of 27 and 38 % in community- and hospital-acquired *E. coli* UTI respectively could not be compared to any other systematic review findings because, to our knowledge, this is the first systematic review and meta-analysis comparing ciprofloxacin resistance in community- and hospital-acquired *E. coli* UTI. However, national data from five WHO regions show at least 50 % resistance to fluoroquinolones (ciprofloxacin, norfloxacin or ofloxacin) in *E. coli* [3]. Data on *E. coli* in the WHO report are from various settings and sources (including blood and urine) hence cannot be directly compared with the results from our systematic review. Another recent review on global fluoroquinolone resistance epidemiology reported a range of 2 to 69 % for fluoroquinolone resistance in uncomplicated community-acquired UTI and up to 98 % in complicated cases, with fluoroquinolone resistance in healthcare associated UTIs ranging from 6 to 62 % [34]. The findings from our systematic review are within the above reported ranges. However, the latter ranges were wide and the data were from a number of different Gram negative uropathogens and not specifically *E. coli* accounting for the higher rates. Available published data show relatively high rates of urinary *E. coli* resistance to ciprofloxacin [35–41] prompting the need for a renewed effort in the further prevention of spread of resistance to this antimicrobial agent.

We found that urinary *E. coli* resistance to ciprofloxacin was higher in the hospital compared to the community setting. Our finding is comparable to individual studies which have assessed urinary *E.coli* resistance to ciprofloxacin in both, hospital and community settings [31, 41–45]. However, often studies do not apply the criterion of 48 h post admission used in our systematic review for identifying hospital acquired UTI [45, 46]. The Canadian national surveillance study (CANWARD), a large population-based study undertaken from 2007 to 2009, further confirms our finding of higher resistance in the hospital setting [47]. Inpatients had a significantly higher urinary *E. coli* resistance to ciprofloxacin. Similar findings were reported by Cullen et al. in Dublin [16]. This is not an unusual finding and may be attributed to the selective pressure resulting from antimicrobial use in hospital settings [47]. Patients in hospital, already acutely ill, become more at risk of developing a resistant infection because of potential immune deficiency and relative high exposure to antimicrobial agents [48]. Furthermore, hospitalized patients are more likely to be exposed to practices that result in cross infection or transmission of organisms. These and other risk factors enable the spread of resistance. This has significant implications for patient care as antimicrobial resistance may lead to treatment failure resulting in death.

The results of our systematic review showed a significant rise in resistance over time in the community setting. This finding is supported by a number of US-based studies investigating antimicrobial resistance trend in outpatients. A fivefold increase (from 3 to 17.1 %) in ciprofloxacin resistance was observed from 2000 to 2010 by Sanchez et al. [17] in comparison with other antibiotics investigated [49]. Our findings are also consistent with Blaettler et al. [6] who found that over a 10 year period (1997–2007), similar to the timeframe for our review, resistance increased significantly for ciprofloxacin from 1.8 to 15.9 % in Switzerland. This increase coincided with a rise in ciprofloxacin use in Switzerland [6]. These findings suggest that with increase in the use of fluoroquinolones generally over time, resistance ciprofloxacin is likely to further increase. It is now known that antimicrobial overuse or misuse is a risk factor for the development of AMR [50]. The specific effect of ciprofloxacin use on the development of its resistance in UTI pathogens is also clearly documented. A recent Irish study involving 72 general practices found higher ciprofloxacin resistance levels (5.5 %) in practices with 10 prescriptions per month compared with resistance levels of 3 % in practices with one prescription per month [51]. Wide spread use of this agent may have thus resulted in a rise in ciprofloxacin resistance. In the Netherlands and United States, an association has also been shown between high fluoroquinolone prescriptions and a rise in bacterial resistance [52, 53]. Furthermore, changes in antimicrobial prescribing practices have been shown to precede changes in resistance rates. A study by Gottesman et al. [54] in Israel found a significant decrease in *E. coli* resistance to ciprofloxacin following a nationwide restriction on ciprofloxacin use. Resistance decreased from 12 % in the pre-intervention period to 9 % in the intervention period. Our results pose a strong argument for the development of more stringent criteria limiting ciprofloxacin use. In addition, other strategies such as adequate surveillance and monitoring, reinforcement of existing infection prevention and control measures as well as new technological advancement will help reduce the widespread problem of antimicrobial resistance [55–57] but these aspects are not within the scope of this paper.

Our finding of a significant rise in resistance over time also has implications for the development of treatment guidelines. The national recommendations for first-choice empiric antibiotic treatment of UTIs vary considerably [5]. In countries like Spain, Taiwan and Turkey, the treatment choice for uncomplicated UTIs are fluoroquinolones [5, 58, 59]. In 2000, fluoroquinolones were prescribed for treatment of uncomplicated UTIs in Switzerland in 64 % of cases [60]. There is concern that resistance to ciprofloxacin resulting from its first-line use may be associated with an increase in multidrug resistance [61]. The most recent IDSA guidelines [9] advise using nitrofurantoin, trimethoprim-sulphamethoxazole, fosfomycin or pivmecillinam for first-line treatment of acute uncomplicated cystitis. Fluoroquinolones should be reserved for important uses other than acute cystitis or used as an alternative only when these recommended agents cannot be used [9]. We recommend that ciprofloxacin should not be used as a first line treatment option for UTIs as continuous increases in resistance to ciprofloxacin further weaken the effectiveness of this drug.

Additional findings from the meta-analysis showed that resistance was significantly higher in developing countries compared to developed countries. A major factor accounting for this difference is the use of over the counter or non-prescription antibiotics which occur commonly in developing countries [62, 63]. Although this review did not directly consider antimicrobial resistance in relation to prescribing for the included studies, evidence shows that over the counter or non-prescription use results in unnecessary and excessive use of antibiotics. Some of the included studies in our review clearly state that there are no restrictions for over the counter prescribing of antimicrobials within their countries [29, 64–73]. A recent systematic review investigating global non-prescription antimicrobial use found that resistance was common in communities with frequent non-prescription antimicrobial use [74]. Non-prescription use was highest in Africa, Asia and Middle East at 100, 58 and 39 % respectively [74]. In our review, further analyses by region showed that Asia had the highest pooled resistance to ciprofloxacin with a significantly higher resistance in Africa, Asia and Middle East combined compared with Europe and the Americas. Our finding is supported by a recent paper by Dalhoff [75] reporting that fluoroquinolone resistance was highest in the Asia-Pacific region and moderate to low in Europe and North America. Furthermore, there is evidence to show that countries that have developed control policies to regulate non-prescription use have seen a decrease in antimicrobial use and resistance rates [74]. Based on our findings, we therefore emphasize the need for the development of policies restricting over the counter antimicrobial use in countries that do not have such policies thereby contributing to the prevention of patient morbidity and mortality associated with resistant infections. It is noteworthy to mention that another important factor contributing to antimicrobial resistance is the use of antibiotics in livestock for growth promotion [76]. Extensive antimicrobial use in food animal production has been associated with antimicrobial resistance globally [76]. This has considerable implications for human health with the need to protect the efficacy of these antimicrobials to ensure their effectiveness for the treatment of humans.

A large variation in ciprofloxacin resistance was found in studies reporting on community-acquired UTI. This variation highlights the significance of local resistance monitoring to guide the development of local antibiotic guidelines. The random effects meta-regression model confirmed that a number of factors significantly accounted for the variations in ciprofloxacin resistance. These include economy (developed and developing), Asia as a region, year of study, studies including only children and studies with a high risk of bias. The first three factors have been discussed in detail in the preceding paragraphs. We found that resistance was lower in studies involving only children. This finding is in line with a number of studies which have compared resistance in adults and children showing significantly higher ciprofloxacin resistance in adults compared to children [77, 78]. Increased age has also been shown to be significantly associated with ciprofloxacin resistance [6, 47]. Given that children are less exposed to antimicrobials with limited ciprofloxacin use in the paediatric age group, this finding is expected [77–79]. Although the importance of intrafamilial cross-infection of resistant pathogens is yet to be confirmed, it has been suggested that fluoroquinolone resistance may to some extent be dependent on cross-infection with transfer from adults to children [78]. Given this assumption, it is necessary to also monitor resistance levels in children to prevent further resistance development in this vulnerable age group. Other likely causes of higher resistance in adults may be the greater likelihood of comorbidities with more frequent contact with healthcare settings [47]. The last factor found to account for heterogeneity between studies was high risk of bias. Most of the studies included in the review were found to have a high risk of bias as assessed using the NOS scale. These studies lacked methodological rigour including absence of the inclusion of possible confounding factors (such as age, sex and previous use of an antimicrobial) in the design and analysis of the studies. The poor reporting of observational studies poses limitations for conducting meta-analysis of these studies. Better presentation of definitions would enable inclusion in systematic reviews of some categories that had to be excluded in this review. Observational studies are more prone to confounding bias [80] further emphasizing the need for adherence to reporting guidelines such as such as that based on the Strengthening the Reporting of Observational Studies in Epidemiology (STROBE) Statement [81] to ensure clear and comprehensive reporting prior to publication acceptance. The poor quality of many studies initially retrieved for this review resulted in a large number being excluded. Therefore the information provided in this systematic review and meta-analysis of 54 observational studies may not sufficiently address ciprofloxacin resistance globally but may provide satisfactory evidence to inform future interventions.

In addition, this systematic review highlights the weaknesses in the quality of antimicrobial resistance data that are being collected in various regions. These weaknesses have implications for development of effective surveillance systems to monitor resistance globally and strategies to prevent further resistance development. The need for the implementation of national and global surveillance systems to detect and continuously monitor AMR cannot be overemphasized. These systems would enable prospective studies to be conducted and would play a major role in curtailing the widespread effect of antimicrobial resistance and help healthcare providers in deciding on the most appropriate empirical therapy for UTI to ensure proper management of patients. Governments need to put in place policies to restrict over the counter use and inappropriate prescribing of ciprofloxacin and other antimicrobials to prevent further development of resistance.

### Strengths and limitations

**Fig. 6:**
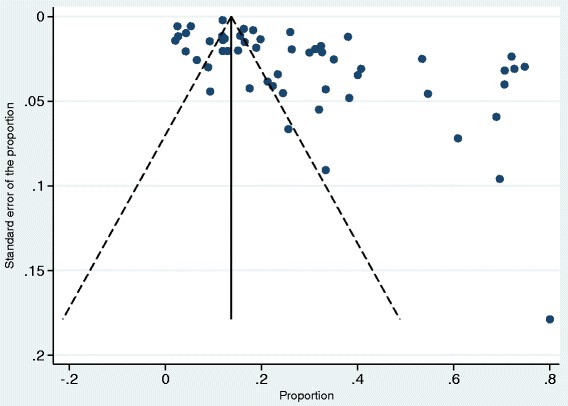
Funnel plot of studies included in meta-analysis

There are a number of notable strengths to our review. To our knowledge, this is the first systematic review to compare the overall prevalence of ciprofloxacin resistance in community- and hospital-acquired *E. coli* UTI. We undertook a comprehensive literature search process to identify and screen articles against eligibility criteria. Given that generic versions of ciprofloxacin were first marketed at different times in various countries, our choice of 2004 as the start date was therefore made on the basis of changes in the epidemiology of antimicrobial resistant pathogens which had resulted in changes to treatment regimens. A further strength of this systematic review is the development of a peer reviewed, registered protocol prior to undertaking the review. For studies to be included in the review, they were restricted to those that used a standard laboratory UTI criterion of ≥10^5^ cfu/mL as recommended by the CDC. Although applying the internationally recognised CDC criteria may definitely be considered a strength as it ensures the quality and uniformity of included studies, this criterion limited the number of hospital-acquired UTI studies included in our systematic review. Despite this, resistance was still found to be higher in the hospital setting compared to the community setting similar to published studies. While lower counts of uropathogens are relevant for acute episodes of uncomplicated cystitis, the use of different colony counts makes comparison of data between studies difficult. Including all urinary *E.coli* isolates was considered but not done because this existing surveillance criterion (≥10^5^ cfu/mL and 48 h cut off) is usually applied to defining infections not isolates. Also, including all isolates carries the risk of including duplicates. This approach poses some degree of ascertainment bias as our systematic review focuses on laboratory identified UTIs which may not only underestimate the total number of UTIs but also lead to selection of samples from complicated cases thereby overestimating resistance. Another limitation is the wide variation of resistance estimates between studies and the inclusion of studies having substantial clinical and methodological heterogeneity. Visual inspection of the funnel plot (Fig. 6) showed asymmetry suggesting evidence of publication bias, with studies reporting high resistance rates being more likely to be published posing a limitation to this review. Also, the quality and risk of bias of some of the studies included in the review were assessed as high. These limitations were addressed by undertaking a random effects meta-analysis with subsequent subgroup analyses and random effects meta-regression to explain the sources of heterogeneity. For studies in which the design was not stated, the review authors faced difficulties in categorising such studies hence some of these studies were grouped as non-classifiable. These studies did not provide clear and explicit information on the methods used for conducting the studies. This emphasizes the need for implementation and adherence to clear reporting standards prior to publication of papers. Furthermore, in some included studies, adjustments were not made for important confounding factors relevant to antimicrobial resistance such as antibiotic use and patient demographics including age and sex. For this systematic review, studies on samples obtained from emergency department (ED) patients were classified as community-acquired samples. Included papers did not provide any information on whether some of these patients may have returned from a recent hospitalisation and represented to the ED. Ideally, these should be considered as hospital-acquired infections as some of these patients may have been discharged in the previous 48 h. For the purpose of this review and to overcome inherent variations in how individual studies have defined these patients, we classified all papers reporting on ED patients as community-acquired UTI studies. It was not possible to determine the potential effect of samples obtained from nursing home or residential aged care studies on the pooled resistance because this participant group did not meet the inclusion criteria for analysis. Furthermore, classification of this setting as hospital or community remains controversial. Finally, validity issues may have arisen from the use of different antimicrobial susceptibility test and interpretation methods with differing breakpoints which tend to change over the years. To date, there is still no worldwide consensus on the most suitable antimicrobial susceptibility testing method with the fact that various countries and even laboratories within the same country use different tests and interpretative criteria. Subgroup analysis for AST method was considered but not done because almost all studies used the disk diffusion method and CLSI criteria.

## Conclusions

Ciprofloxacin resistance in *E. coli* UTI is increasing. The use of this antimicrobial agent as empirical therapy for UTI should be reconsidered and efforts should be made to limit its use to clinical conditions for which there are clear therapeutic indications. Policy restrictions on ciprofloxacin use need to be developed and enforced especially in developing countries that are yet to have such policies put in place. Further research is needed to describe ciprofloxacin resistance in hospital-acquired *E. coli* UTI using widely accepted definitions.

### Availability of Data and Materials

Data supporting the findings of this systematic review are available upon request from the corresponding author.

## Additional files

Additional file 1:
**Search strategy by database.** Search of EMBASE, CINHAL, Scopus, PubMed, MEDLINE and COCHRANE. (PDF 58 kb) Additional file 2:
**Subgroup analyses of pooled ciprofloxacin resistance in hospital setting.** Results of subgroup analyses for studies reporting on hospital acquired *E. coli* UTI. (PDF 12 kb) Additional file 3:
**Subgroup analyses of pooled ciprofloxacin resistance by setting.** Results of subgroup analyses comparing the pooled resistance within each subgroup examined for both community and hospital settings. (PDF 36 kb)
