# Supplementary material for: Ciprofloxacin resistance in community- and hospital-acquired Escherichia coli urinary tract infections: a systematic review and meta-analysis of observational studies
Source: BMC Infect Dis. 2015 Nov 25;15:545. doi: 10.1186/s12879-015-1282-4 (PMC4660780; doi:10.1186/s12879-015-1282-4)
Supplement: Additional file 1: — Search strategy by database. Search of EMBASE, CINHAL, Scopus, PubMed, MEDLINE and COCHRANE. (PDF 58 kb) [file 12879_2015_1282_MOESM1_ESM.pdf]

ADDITIONAL FILES

Additional file 1: Search strategy by database

EMBASE Search strategy 1: Keywords only

#	Searches	Results
1	resistance.mp.	798263
2	urinary tract infection.mp.	80151
3	Escherichia coli.mp.	358062
4	1 and 2 and 3	3709
5	4 and 2004:2014.(sa_year).	2423

EMBASE Search strategy 2: Keywords and subject headings

#	Searches	Results
1	antibiotic resistan*.mp. [mp=title, abstract, subject headings, heading word, drug trade name, original title, device manufacturer, drug manufacturer, device trade name, keyword]	123069
2	antimicrobial resistan*.mp. [mp=title, abstract, subject headings, heading word, drug trade name, original title, device manufacturer, drug manufacturer, device trade name, keyword]	12903
3	drug resistan*.mp. [mp=title, abstract, subject headings, heading word, drug trade name, original title, device manufacturer, drug manufacturer, device trade name, keyword]	114373
4	bacterial resistan*.mp. [mp=title, abstract, subject headings, heading word, drug trade name, original title, device manufacturer, drug manufacturer, device trade name, keyword]	5524
5	antibiotic resistance/	114397
6	drug resistance/	58485
7	1 or 2 or 3 or 4 or 5 or 6	225773
8	urinary tract infection*.mp. [mp=title, abstract, subject headings, heading word, drug trade name, original title, device manufacturer, drug manufacturer, device trade name, keyword]	83408
9	uti.mp. [mp=title, abstract, subject headings, heading word, drug trade name, original title, device manufacturer, drug manufacturer, device trade name, keyword]	9689
10	bacteriuria.mp. [mp=title, abstract, subject headings, heading word, drug trade name, original title, device manufacturer, drug manufacturer, device trade name, keyword]	10917
11	pyuria.mp. [mp=title, abstract, subject headings, heading word, drug trade name, original title, device manufacturer, drug manufacturer, device trade name, keyword]	3890
12	urinary tract infection/	75810
13	bacteriuria/	8487
14	pyuria/	3150
15	8 or 9 or 10 or 11 or 12 or 13 or 14	91929
16	Escherichia coli.mp. [mp=title, abstract, subject headings, heading word, drug trade name, original title, device manufacturer, drug manufacturer, device trade name, keyword]	358062
17	e coli.mp. [mp=title, abstract, subject headings, heading word, drug trade name, original title, device manufacturer, drug manufacturer,	125639

	device trade name, keyword]	
18	Escherichia coli/	294320
19	16 or 17 or 18	371005
20	7 and 15 and 19	3284
21	20 and 2004:2014.(sa_year).	2160

CINAHL Search strategy 1: Keywords only

#	Searches	Results
1	resistance	55753
2	urinary tract infection	7192
3	escherichia coli	4970
4	1 AND 2 AND 3	235
5	4 Limiters-Published date 20040101-20141231	208

CINAHL Search strategy 2: Keywords and subject headings

#	Searches	Results
1	antibiotic resistan*	4759
2	antimicrobial resistan*	3316
3	drug resistan*	33393
4	bacterial resistan*	6765
5	(MH “Drug Resistance, Microbial”)	12164
6	(MH “Drug Resistance”)	4838
7	1 OR 2 OR 3 OR 4 OR 5 OR 6	36416
8	urinary tract infection*	7781
9	uti	1081
10	bacteriuria	740
11	pyuria	129
12	(MH “Urinary Tract Infections”)	5924
13	(MH “Bacteriuria”)	518
14	8 OR 9 OR 10 OR 11 OR 12 OR 13	8169
15	escherichia coli	4970
16	e coli	1428
17	(MH “Escherichia Coli”)	2722
18	15 OR 16 OR 17	5191
19	7 AND 14 AND 18	270
20	19; Limiters – Published Date: 20040101-20141231	236

SCOPUS Search strategy 1: Keywords only

#	Searches	Results
1	TITLE-ABS-KEY (resistance)	1,406,458
2	TITLE-ABS-KEY (urinary tract infection)	87,237
3	TITLE-ABS-KEY (escherichia coli)	411,648
4	(TITLE-ABS-KEY (resistance)) AND (TITLE-ABS-KEY (urinary tract infections)) AND (TITLE-ABS-KEY (escherichia coli))	4,353
5	(TITLE-ABS-KEY (resistance)) AND (TITLE-ABS-KEY (urinary tract infections)) AND (TITLE-ABS-KEY (escherichia coli)) AND (LIMIT-TO (PUBYEAR , 2014-2004)	2472

SCOPUS Search strategy 2: Keywords and subject headings

#	Searches	Results
1	TITLE-ABS-KEY (antibiotic resistan*)	186424
2	TITLE-ABS-KEY (antimicrobial resistan*)	56777
3	TITLE-ABS-KEY (drug resistan*)	507251
4	TITLE-ABS-KEY (bacterial resistan*)	181814
5	1 OR 2 OR 3 OR 4	597338
6	TITLE-ABS-KEY (urinary tract infection)	87237
7	TITLE-ABS-KEY (uti)	7656
8	TITLE-ABS-KEY (bacteriuria)	11141
9	TITLE-ABS-KEY (pyuria)	3187
10	6 OR 7 OR 8 OR 9	94035
11	TITLE-ABS-KEY (escherichia coli)	411648
12	TITLE-ABS-KEY (e coli)	168002
13	11 OR 12	429893
14	5 AND 10 AND 13	5184
15	14 AND (LIMIT-TO (PUBYEAR , 2014-2004)	2777

PubMed Search strategy 1: Keywords only

#	Searches	Results
1	Search resistance	610335
2	Search urinary tract infection	54,476
3	Search escherichia coli	318047
4	Search ((resistance) AND urinary tract infection) AND escherichia coli	2,416
5	4 AND Filters: Publication date from 2004/01/01 to 2014/12/31	1243

PubMed Search strategy 2: Keywords and subject headings

#	Searches	Results
1	Search antibiotic resistance	146589
2	Search antimicrobial resistance	165154
3	Search bacterial resistance	116736
4	Search drug resistance	375235
5	Search "Drug Resistance, Microbial"[Mesh]	125068
6	Search "Drug Resistance, Bacterial"[Mesh]	60535
7	1 OR 2 OR 3 OR 4 OR 5 OR 6	409413
8	Search urinary tract infection	54469
9	Search uti	5705
10	Search bacteriuria	8749
11	Search pyuria	1976
12	Search "Urinary Tract Infections"[Mesh]	38550
13	Search "Bacteriuria"[Mesh]	6931
14	Search "Pyuria"[Mesh]	906
15	8 OR 9 OR 10 OR 11 OR 12 OR 13 OR 14	56345
16	Search escherichia coli	318008
17	Search e coli	333732
18	Search "Escherichia coli"[Mesh]	232783
19	16 OR 17 OR 18	333732
20	7 AND 15 AND 19	2623

21	20 AND Filters: Publication date from 2004/01/01 to 2014/12/31	1301

MEDLINE Search strategy 1: Keywords only

#	Searches	Results
1	resistance.mp.	574974
2	urinary tract infections.mp.	37313
3	Escherichia coli.mp.	311661
4	1 and 2 and 3	1923
5	4 and 2004:2014. (sa_year)	964

MEDLINE Search strategy 2: Keywords and subject headings

#	Searches	Results
1	antibiotic resistan*.mp. [mp=title, abstract, original title, name of substance word, subject heading word, keyword heading word, protocol supplementary concept word, rare disease supplementary concept word, unique identifier]	19772
2	antimicrobial resistan*.mp. [mp=title, abstract, original title, name of substance word, subject heading word, keyword heading word, protocol supplementary concept word, rare disease supplementary concept word, unique identifier]	8841
3	drug resistan*.mp. [mp=title, abstract, original title, name of substance word, subject heading word, keyword heading word, protocol supplementary concept word, rare disease supplementary concept word, unique identifier]	197925
4	bacterial resistan*.mp. [mp=title, abstract, original title, name of substance word, subject heading word, keyword heading word, protocol supplementary concept word, rare disease supplementary concept word, unique identifier]	3438
5	Drug Resistance, Microbial/	54415
6	Drug Resistance/	39696
7	1 or 2 or 3 or 4 or 5 or 6	208986
8	urinary tract infection*.mp. [mp=title, abstract, original title, name of substance word, subject heading word, keyword heading word, protocol supplementary concept word, rare disease supplementary concept word, unique identifier]	43114
9	uti.mp. [mp=title, abstract, original title, name of substance word, subject heading word, keyword heading word, protocol supplementary concept word, rare disease supplementary concept word, unique identifier]	5172
10	bacteriuria.mp. [mp=title, abstract, original title, name of substance word, subject heading word, keyword heading word, protocol supplementary concept word, rare disease supplementary concept word, unique identifier]	8646
11	pyuria.mp. [mp=title, abstract, original title, name of substance word, subject heading word, keyword heading word, protocol supplementary concept word, rare disease supplementary concept word, unique identifier]	1890
12	Urinary Tract Infections/	32461
13	Bacteriuria/	7048

14	Pyuria/	925
15	8 or 9 or 10 or 11 or 12 or 13 or 14	49102
16	Escherichia coli.mp. [mp=title, abstract, original title, name of substance word, subject heading word, keyword heading word, protocol supplementary concept word, rare disease supplementary concept word, unique identifier]	311661
17	e coli.mp. [mp=title, abstract, original title, name of substance word, subject heading word, keyword heading word, protocol supplementary concept word, rare disease supplementary concept word, unique identifier]	120235
18	Escherichia coli/	227408
19	16 or 17 or 18	324796
20	7 and 15 and 19	1886
21	20 and 2004:2014.(sa_year).	933

COCHRANE Search strategy

Cochrane Database of Systematic Reviews

#	Searches	Cochrane reviews
1	resistance in Title, Abstract, Keywords (Word variations have been searched)	263
2	urinary tract infection in Title, Abstract, Keywords (Word variations have been searched)	77
3	Escherichia coli in Title, Abstract, Keywords (Word variations have been searched)	3
4	1 and 2 and 3	1
5	4 and Publication Year from 2004 to 2014 (Word variations have been searched)	1
