# Supplementary material for: Ciprofloxacin resistance in community- and hospital-acquired Escherichia coli urinary tract infections: a systematic review and meta-analysis of observational studies
Source: BMC Infect Dis. 2015 Nov 25;15:545. doi: 10.1186/s12879-015-1282-4 (PMC4660780; doi:10.1186/s12879-015-1282-4)
Supplement: Additional file 2: — Subgroup analyses of pooled ciprofloxacin resistance in hospital setting. Results of subgroup analyses for studies reporting on hospital acquired E. coli UTI. (PDF 12 kb) [file 12879_2015_1282_MOESM2_ESM.pdf]

**Additional file 2: Subgroup analyses of pooled ciprofloxacin resistance in hospital setting**

| Subgroup | | Hospital setting N=3 Pooled resistance | *P* value* |
|---|---|---|---|
| Region | Middle East n=1 study | 0.400** | 0.880 |
| | North America n=1 study | 0.407** | |
| Economy | Developed n=1 study | 0.407 | 0.880 |
| | Developing n=1 study | 0.400 | |
| UTI symptoms | Symptomatic and asymptomatic patients n=1 study | 0.380 | 0.356 |
| | Symptomatic patients only n=2 studies | 0.404 | |

*****Comparing pooled resistance for difference in subgroup in hospital setting
n=number of studies reporting on hospital acquired UTI
**Middle East only; North America only
