# Supplementary material for: Ciprofloxacin resistance in community- and hospital-acquired Escherichia coli urinary tract infections: a systematic review and meta-analysis of observational studies
Source: BMC Infect Dis. 2015 Nov 25;15:545. doi: 10.1186/s12879-015-1282-4 (PMC4660780; doi:10.1186/s12879-015-1282-4)
Supplement: Additional file 3: — Subgroup analyses of pooled ciprofloxacin resistance by setting. Results of subgroup analyses comparing the pooled resistance within each subgroup examined for both community and hospital settings. (PDF 36 kb) [file 12879_2015_1282_MOESM3_ESM.pdf]

Additional file 3: Subgroup analyses of pooled ciprofloxacin resistance by setting

Subgroup		Community Setting N=51	Hospital setting N=3	*P* value*
		Pooled resistance	Pooled resistance	
Risk of bias	Low and unclear C=28 studies H= 0 study	0.221	-	-
	High C=23 studies H=3 studies	0.337 (10262)	0.385	<0.0001
Study duration	≤12 months C=25 studies H=0 study	0.323	-	-
	>12months C=24 studies H=3 studies	0.219 (34328)	0.385	<0.0001
Study design	Cross sectional C=40 studies H=3 studies	0.271 (36909)	0.385	<0.0001
	Cohort C=5 studies H=0 study	0.287	-	-
	Case control C=2 studies H=0 study	0.224	-	-
Economy	Developed C=16 studies H=1 study	0.141 (12996)	0.407	<0.0001
	Developing C=35 studies H=1 study	0.345 (32064)	0.400	0.103
Region	Africa C=7 studies H=0 study	0.203	-	-
	Asia and Middle East C=22 studies H=1 study	0.390 (8866)	0.400**	0.774
	Europe C=13 studies H=0 study	0.156	-	-
	North and South America	0.207 (24871)	0.407**	<0.0001

	C=9 studies<br>H=1 study			
Age group	Adults and children ‡<br>C=24 studies<br>H=2 studies	0.265 (33090)	0.382	<0.0001
	Adults only<br>C=19 studies<br>H=0 study	0.302	-	-
UTI symptoms	Symptomatic and asymptomatic patients<br>C=11 studies<br>H=1 study	0.185 (27984)	0.380	<0.0001
	Symptomatic patients only<br>C=40 studies<br>H=2 studies	0.295 (17076)	0.404	<0.0001
Overall		0.27	0.38	<0.0001

*Comparing pooled resistance for difference in subgroup in community and hospital settings

‡ Studies reporting resistance in adults and children or children only

C= number of studies reporting on community acquired UTI

H= number of studies reporting on hospital acquired UTI

**Middle East only; North America only
